# Supplementary material for: TRex, a fast multi-animal tracking system with markerless identification, and 2D estimation of posture and visual fields
Source: eLife. 2021 Feb 26;10:e64000. doi: 10.7554/eLife.64000 (PMC8096434; doi:10.7554/eLife.64000)
Supplement: Transparent reporting form [file elife-64000-transrepform.pdf]

eLIFE
Westbrook Centre, Milton Road
Cambridge CB4 1YG
UK
P 01223 855340
W elifesciences.org
T @elife

# *eLife's* transparent reporting form

We encourage authors to provide detailed information *within their submission* to facilitate the interpretation and replication of experiments. Authors can upload supporting documentation to indicate the use of appropriate reporting guidelines for health-related research (see EQUATOR Network), life science research (see the BioSharing Information Resource), or the ARRIVE guidelines for reporting work involving animal research. Where applicable, authors should refer to any relevant reporting standards documents in this form.

If you have any questions, please consult our Journal Policies and/or contact us: editorial@elifesciences.org.

**Sample-size estimation**

- You should state whether an appropriate sample size was computed when the study was being designed
- You should state the statistical method of sample size computation and any required assumptions
- If no explicit power analysis was used, you should describe how you decided what sample (replicate) size (number) to use

Please outline where this information can be found within the submission (e.g., sections or figure legends), or explain why this information doesn't apply to your submission:

Previously published (Pérez-Escudero et al. 2014) and unpublished video data has been used in the evaluation of TRex. Unpublished material was acquired under the ethical licences 35-9185.81/G-17/162, 35-9185.81/G-17/88 and 35-9185.81/G-16/116 granted by the Regional Council of the State of Baden-Württemberg, Freiburg, Germany, to the Max Planck Institute of Animal Behavior in accordance with the German Animal Welfare Act (TierSchG) and the Regulation for the Protection of Animals Used for Experimental or Other Scientific Purposes (Animal Welfare Regulation Governing Experimental Animals - TierSchVersV). Appropriate sample sizes metrics for the evaluation of the tool performance are provided in the respective figure legends. These are mostly means and standard deviations. As this study evaluates the performance of TRex with respect to video data a power analysis does not apply.

**Replicates**

- You should report how often each experiment was performed
- You should include a definition of biological versus technical replication
- The data obtained should be provided and sufficient information should be provided to indicate the number of independent biological and/or technical replicates
- If you encountered any outliers, you should describe how these were handled
- Criteria for exclusion/inclusion of data should be clearly stated
- High-throughput sequence data should be uploaded before submission, with a private link for reviewers provided (these are available from both GEO and ArrayExpress)

Please outline where this information can be found within the submission (e.g., sections or figure legends), or explain why this information doesn't apply to your submission:

eLife Sciences Publications, Ltd is a limited liability non-profit non-stock corporation incorporated in the State of Delaware, USA, with company number 5030732, and is registered in the UK with company number FC030576 and branch number BR015634 at the address Westbrook Centre, Milton Road Cambridge CB4 1YG, UK | March 2019

TRex was run 5 times for videos 7-16 with visual identification, once for each speed measurement using all videos (with and without posture for each of the different tracking modes 'tree-based', 'approximate' and 'hungarian'). Conversion was performed 3 times for all videos to measure conversion speed when tracking at the same time, and also 3 times with live-tracking disabled. idtracker.ai was run 3 times for Videos 7-16, except Videos 9 and 10 which we were unable to track using idtracker.ai despite considerable effort (see main text for details). We did not exclude any results, all trials were run consecutively on the same computer, using the same script (with the exception of Fig.5, which is unrelated and was produced separately).

**Statistical reporting**

- Statistical analysis methods should be described and justified
- Raw data should be presented in figures whenever informative to do so (typically when N per group is less than 10)
- For each experiment, you should identify the statistical tests used, exact values of N, definitions of center, methods of multiple test correction, and dispersion and precision measures (e.g., mean, median, SD, SEM, confidence intervals; and, for the major substantive results, a measure of effect size (e.g., Pearson's r, Cohen's d)
- Report exact p-values wherever possible alongside the summary statistics and 95% confidence intervals. These should be reported for all key questions and not only when the p-value is less than 0.05.

Please outline where this information can be found within the submission (e.g., sections or figure legends), or explain why this information doesn't apply to your submission:

Necessary information can be found in Figure Legends, or Jupyter Notebooks (released as part of the Reproduction Dataset at https://dx.doi.org/10.17617/3.4y)

(For large datasets, or papers with a very large number of statistical tests, you may upload a single table file with tests, Ns, etc., with reference to sections in the manuscript.)

**Group allocation**

- Indicate how samples were allocated into experimental groups (in the case of clinical studies, please specify allocation to treatment method); if randomization was used, please also state if restricted randomization was applied
- Indicate if masking was used during group allocation, data collection and/or data analysis

Please outline where this information can be found within the submission (e.g., sections or figure legends), or explain why this information doesn't apply to your submission:

RAW videos were selected to provide a wide variety of group sizes, organisms, and camera models. Videos used for similarity measurements (between idtracker.ai and TRex) were selected based on whether we could configure idtracker.ai to track them successfully. TRex' visual identification was used on all videos with <= 100 individuals.

**Additional data files ("source data")**

- We encourage you to upload relevant additional data files, such as numerical data that are represented as a graph in a figure, or as a summary table

- Where provided, these should be in the most useful format, and they can be uploaded as "Source data" files linked to a main figure or table
- Include model definition files including the full list of parameters used
- Include code used for data analysis (e.g., R, MatLab)
- Avoid stating that data files are "available upon request"

Please indicate the figures or tables for which source data files have been provided:

All files required to reproduce the results in this paper are available from https://dx.doi.org/10.17617/3.4y (see README.txt in that repository)
